# Thermodynamic Aspects and Reprogramming Cellular Energy Metabolism during the Fibrosis Process

**DOI:** 10.3390/ijms18122537

**Published:** 2017-11-27

**Authors:** Alexandre Vallée, Yves Lecarpentier, Jean-Noël Vallée

**Affiliations:** 1 Laboratory of Mathematics and Applications (LMA), DACTIM, UMR CNRS 7348, CHU de Poitiers and University of Poitiers, 86021 Poitiers, France; valleejn@gmail.com; 2 Centre de Recherche Clinique, Grand Hôpital de l’Est Francilien (GHEF), 77100 Meaux, France; yves.c.lecarpentier@gmail.com; 3 CHU Amiens Picardie, University of Picardie Jules Verne (UPJV), 80025 Amiens, France

**Keywords:** aerobic glycolysis, canonical WNT/β-catenin pathway, circadian rhythms, fibrosis, TGF-β, Warburg effect

## Abstract

Fibrosis is characterized by fibroblast proliferation and fibroblast differentiation into myofibroblasts, which generate a relaxation-free contraction mechanism associated with excessive collagen synthesis in the extracellular matrix, which promotes irreversible tissue retraction evolving towards fibrosis. From a thermodynamic point of view, the mechanisms leading to fibrosis are irreversible processes that can occur through changing the entropy production rate. The thermodynamic behaviors of metabolic enzymes involved in fibrosis are modified by the dysregulation of both transforming growth factor β (TGF-β) signaling and the canonical WNT/β-catenin pathway, leading to aerobic glycolysis, called the Warburg effect. Molecular signaling pathways leading to fibrosis are considered dissipative structures that exchange energy or matter with their environment far from the thermodynamic equilibrium. The myofibroblastic cells arise from exergonic processes by switching the core metabolism from oxidative phosphorylation to glycolysis, which generates energy and reprograms cellular energy metabolism to induce the process of myofibroblast differentiation. Circadian rhythms are far-from-equilibrium thermodynamic processes. They directly participate in regulating the TGF-β and WNT/β-catenin pathways involved in energetic dysregulation and enabling fibrosis. The present review focusses on the thermodynamic implications of the reprogramming of cellular energy metabolism, leading to fibroblast differentiation into myofibroblasts through the positive interplay between TGF-β and WNT/β-catenin pathways underlying in fibrosis.

## 1. Introduction

Fibrosis is an irreversible and non-physiological scarring process associated with inflammation and increased extracellular matrix (ECM) deposition contributing to tissue damage. Fibrosis is characterized by fibroblast proliferation, fibroblast differentiation into myofibroblasts, and synthesis of ECM including collagen and proteoglycans. Several causes are known for fibrosis, such as infection, alcohol, drug, genetic alterations, radiation, or environmental factors [1]. Fibrosis is not restricted to some tissues but can occur in all organs and tumors [2,3,4,5]. A common pathogenic pattern exists in which fibroblasts are ECM producers [1]. Fibroblast proliferation increases in fibrosis, and myofibroblasts are the differentiated form of fibroblasts [6]. Myofibroblasts can derive from bone-marrow-derived fibrocytes, pericytes, vascular smooth muscle cells, or tissue-resident fibroblasts [7,8,9].

From a thermodynamic point of view, myofibroblastic cells arise from exergonic processes and generate heat within their surroundings. The mechanisms leading to the development of fibrosis are similar to many irreversible processes that can occur due to a change in the entropy production rate [10,11,12,13].

Several molecular mechanisms can induce and develop the fibrotic process. In fibrosis, thermodynamic behaviors of metabolic enzymes are modified by the dysregulation of both transforming growth factor β (TGF-β) signaling and the canonical WNT/β-catenin pathway [14,15].

Upregulation of TGF-β1 activates lactate dehydrogenase (LDH) expression, leading to lactate production, and induces fibroblast differentiation into myofibroblast and excessive collagen deposition [16,17]. Myofibroblasts are non-muscle contractile cells containing alpha-smooth muscle actin (α-SMA), and are characterized by a dramatic slowness of their contractile kinetics [18,19].

Upregulation of the canonical WNT/β-catenin pathway activates pyruvate dehydrogenase kinase-1 (PDK-1), which decreases pyruvate dehydrogenase complex (PDH) activity. Upregulation of canonical WNT/β-catenin pathway also stimulates Lactate Dehydrogenase A (LDH-A) and monocarboxylate lactate transporter-1 (MCT-1) [20]. Thus, WNT/β-catenin activation decreases the conversion of pyruvate into acetyl-coenzyme A (acetyl-CoA) in mitochondria and its entry into the tricarboxylic acid (TCA) cycle. At this step, a major part of cytosolic pyruvate is converted into lactate as the primary alternative of oxidative phosphorylation despite the availability of oxygen, a phenomenon called aerobic glycolysis or the Warburg effect [21].

TGF-β1 and WNT/β-catenin pathways leading to fibrosis can be considered dissipative structures that exchange energy or matter with their environment [22]. These pathological processes operate as far-from-equilibrium open systems. Circadian rhythms are also far-from-equilibrium thermodynamic processes [11,12].

We focus this review on the thermodynamic implications in the reprogramming of cellular energy metabolism, enabling fibroblast differentiation into myofibroblasts through the positive interplay of the molecular signaling pathways TGF-β and WNT/β-catenin underlying the fibrotic process.

## 2. Thermodynamic Aspects of Myofibroblasts

Myofibroblasts are contractile non-muscle cells that exhibit bundles of actin filament containing α-SMA. Myofibroblasts are connected by α-SMA peripheral focal adhesions and gap junctions in the granulation tissue [23]. Fibroblasts become protomyofibroblasts. Protomyofibroblasts synthetize ECM, collagen, and fibronectin for their differentiation into myofibroblasts [24] containing α-SMA and non-muscle myosin II (NMII), responsible for the retractile role of myofibroblasts [25].

Differentiation of fibroblasts into myofibroblasts requires the participation of physical and chemical factors such as an increase in tissue stiffness [26,27] and the activated TGF-β1 association with the fibronectin extra domain A (EDA) [24,28]. The activated TGF-β1-promoted α-SMA increases the contractile property of myofibroblasts [29]. Transmission of the contractile force generated by α-SMA and the molecular motor myosin are performed by ECM through focal adhesions containing transmembrane integrin [30]. TGF-β1 activation in ECM is an integrin-dependent process [31].

The molecular motor of myofibroblasts is the non-muscle myosin IIA (NMIIA) [32]. NMIIA is characterized by three pairs of chains, including two 230 kDa heavy chains, two 20 kDa regulatory light chains (RLCs) that stimulate the activity of NMII, and two 17 kDa essential light chains (ELCs) that stabilize the structure of heavy chains. NMII is responsible for cell polarity, cell–cell adhesion, and cell migration.

Myosin filaments link actin filaments in thick contractile actomyosin bundles like stress fibers. The NMIIA molecules assemble into bipolar filaments. A tilted state of the myosin head enables a conformational change that moves actin filaments in an anti-parallel manner. The actin–myosin cross-bridge (CB) cycle of NMIIA is overall like smooth and striated muscle myosin. An ATP molecule binds the NMIIA-ATPase site on the head of the myosin, which enables the dissociation of actin from the NMIIA head, and the ATP hydrolysis. Subsequently, NMIIA binds to actin. The power stroke then occurs with a tilt of the NMIIA head, generating a CB single force with a displacement of few nanometers, and then releasing ADP from the acto-NMIIA complex, as the final stage of a CB cycle. A new CB cycle starts when a new ATP molecule dissociates actin from the myosin head.

From a thermodynamic point of view, contractile tissues are open biological systems not in equilibrium state through the exchanges of energy and matter with their surroundings, and can operate either close to or far away from thermodynamic equilibrium [10,33]. In the physiological state, muscular and non-muscular contractile tissues, which have the ability to contract and relax, have been shown to be open chemical systems maintained in a near-equilibrium thermodynamic state that operate under a linear regime, evolving to a stationary state in which the entropy production rate is minimal [33].

However, the singularity of the motor molecular NMIIA that characterizes contractile non-muscular myofibroblast cells is its dramatically slow cycle of contractile kinetics compared to that of myosin of contractile muscle cells [18,19]. Indeed, myofibroblasts occur physiologically in organs such as in stem villi of the human placenta during normal pregnancies. In these contractile non-muscle cells of the human placenta, the rate of attachment and detachment of the actin-myosin CB, and the catalytic constant are lower than those of striated or smooth muscles, although, the actin-myosin CB single force of NMIIA is on the same order of magnitude as muscle myosin II (MII) [33]. This singularity confers a particular thermodynamic profile for NMIIA with low thermodynamic flow, low myosin content and ATPase activity of NMIIA, and low thermodynamic entropy production rate compared with contractile muscle tissues [33]. Such a stationary contractile system ensures that numerous contraction–relaxation cycles operate at a near-equilibrium thermodynamic state, mainly through mitochondrial oxidative phosphorylation energy metabolism.

In the fibrosis process, myofibroblasts generate a relaxation-free contraction mechanism associated with excessive collagen synthesis in the ECM that promotes irreversible tissue retraction, evolving towards fibrosis and finally apoptosis of myofibroblasts [33,34,35,36].

The mechanisms leading to fibrosis are like many irreversible processes that can occur due to a change in the entropy production rate. This rate represents a thermodynamic quantity, such as heat production, Gibbs energy, or ionic conductance, and quantifies these irreversible processes [10,11,12,13]. The myofibroblast cells arise from exergonic processes, mainly through glycolysis energy metabolism and generate heat within the surrounding environment.

The molecular signaling pathways leading to the development of fibrosis are considered as dissipative structures that exchange energy or matter with their environment. They operate as far-from-equilibrium open thermodynamic systems under non-linear regime evolving to non-stationary states. Circadian rhythms are also dissipative structures that operate as far-from-equilibrium thermodynamics [11,12]. Moreover, circadian rhythms directly participate in regulating the molecular pathway TGF-β [37] and WNT/β-catenin [38] is involved in the reprogramming of cellular energy metabolism, enabling fibroblast differentiation into myofibroblasts.

## 3. TGF-β1, Reactive Oxygen Species, and the Fibrotic Process

**Figure 1:**
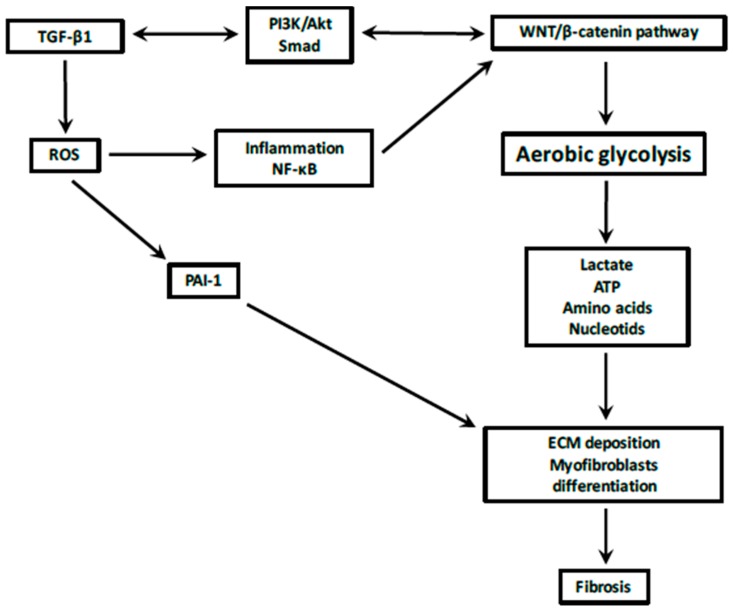
Reprogramming energetic metabolism through the interactions between TGF-β1, Transforming growth factor 1 and WNT/β-catenin pathway during the fibrotic process. PAI-1, Plasminogen Activator Inhibitor 1; NF-κB, NFkappaB; ROS, Reactive Oxygen Species; PI3K, Phosphatidylinositol−4,5-biphosphatase 3-kinase.

The production [39,40,41] and activity [17] of TGF-β1 are stimulated in fibrosis. TGF-β1 activation induce the transformation of fibroblasts into α-SMA-expressing myofibroblasts, and the excessive synthesis of ECM proteins including collagen and metalloproteinases (MMPs) [42,43,44,45,46]. TGF-β1 and Smad pathways interact in myofibroblasts through the phosphorylation of Smad2 and Smad3 by TGF-β receptor 1 (TGF-βR1). TGF-β1 binds TGF-βR2 and their interaction with TGF-βR1 forms a heterotetramer for the Smad2 and Smad3 phosphorylation. Then, Smad2/3 binds Smad4, and the complex thus formed translocates to the nucleus for activation of the Smad binding element (SBE) [47]. TGF-β1 activation also stimulates Smad-independent pathways, such as the phosphatidylinositol 3-kinase/serine/threonine kinase (protein kinase B) (PI3K/Akt) pathway [48]. PI3K/Akt pathway activation is involved in collagen synthesis via TGF-β1 signaling [49,50,51] (Figure 1).

Several studies have shown that reactive oxygen species (ROS) have a major role in the fibrosis process. ROS production has been observed in several fibrotic tissues such as renal fibrosis and fibrotic myocardial infarction [52,53,54,55,56]. ROS production leads to the activation of cytokines and growth factors [57,58,59], and the production of various enzymes, such as NADPH oxidase (named NOX) [60]. The NOX enzyme has a main role in the fibrotic process [61,62]. NOX-derived ROS is associated with fibrotic pathway in several organs, such as lung [63], heart [64], kidney [65], pancreas [66], and liver [67,68,69]. ROS and TGF-β1 are linked and considered major keys to fibrosis [58,70].

**Figure 2:**
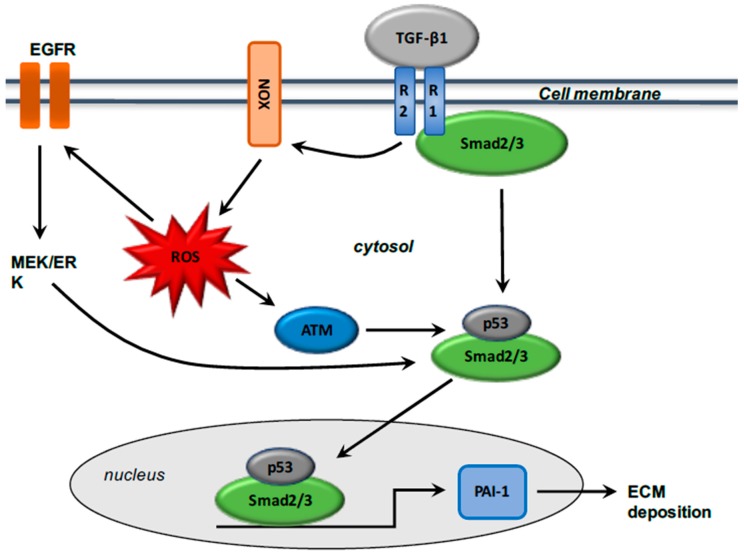
Interactions between Smad and non-Smad pathways with TGF-β1 signaling. TGF-β1 receptor (TGF-βR1) activation, consequent to the ligand TGF-β1 engagement, initiates the activation of Smad2/3 (through the phosphorylation of Smad2 and Smad3 by TGF-βR1), non-Smad (EGFR) mediated downstream pathways (like PAI-1,) and phenotypic modifications (like myofibroblast differentiation and extracellular matrix deposition/fibrosis). TGF-β1, by inducing NOX expression, activates ROS generation, which enhances the initiation of non-Smad (EGFR) and the modulation of Smad (maintenance of Smad phosphorylation). The p53 transcription factor integrates transcriptional contributions from both Smad and non-Smad cascades. ROS generation by TGF-β1 stimulation is crucial for p53 activation (by phosphorylation and acetylation).

ROS production is enhanced by TGF-β1 activation [1], and TGF-β1-mediated NOX4 expression promotes ROS production [64,71,72,73]. NOX expression is upregulated in several models of fibrosis [74]. TGF-β1 stimulates ROS generation by inducing NOX expression [75]. Inhibition of NOX4 induces downregulation of TGF-β1 expression and then diminution of ECM deposition [63,76]. During the fibrotic process, TGF-β1 activated binds Smad2/3 or PI3K signaling and then activates NOX4 expression. NOX4 in turn stimulates ROS production, which activates the proliferation, migration, and differentiation of fibroblasts and induces ECM deposition [1]. TGF-β1, by activating epidermal growth factor receptor (EGFR) signaling, stimulates the expression of the potent profibrotic matricellular plasminogen activator inhibitor-1 (PAI-1), which encodes the structural elements of ECM such as fibronectin and collagen 1 [77,78,79,80]. PAI-1 is one of the most important precursors in tissue inflammation and fibrosis [81], limiting the degradation of ECM and facilitating the accumulation of matrix structural elements [82,83]. TGF-β1-induced PAI-1 expression is involved in ROS signaling, leading to the regulation of strong interactions with Smad and non-Smad axes [84], and then enhances EGFR cascade [85] and TGF-β1 R1-directed Smad2/3 phosphorylation [86]. ROS generation activates c-Src and subsequently the phosphorylation of EGFR, which stimulates the MEK-ERK1/2 cascade, which is an activator of p53 [87] (Figure 2).

Several studies have shown that tumor suppressor p53 is a critical factor in the fibrotic process [88]. Moreover, ROS generation is considered an upstream influence on p53 signaling through the stimulation of ataxia telangiectasia mutated (ATM) [89] in injured tissues [90] and in mouse embryo fibroblasts [91]. TGF-β1 stimulates p53 by phosphorylation and then leads to the activated phosphorylated Smad2/3/p53 complex [88], which binds to PAI-1 [92]. Interactions between p53 and the Smad pathway are crucial for activation of PAI-1 via TGF-β1, suggesting crosstalk between Smad and non-Smad such as p53 and NOX [93].

ROS production and inflammation act in a vicious circle in which inflammation stimulates ROS, which in turn enhance inflammation through activation of the NF-κB pathway [94,95,96]. In parallel, ROS production causes DNA damage in endothelial cells and induces activation of the canonical WNT/β-catenin pathway [97].

## 4. Canonical WNT/β-Catenin Pathway during the Fibrotic Process

The canonical WNT/β-catenin pathway is upregulated in fibrotic tissues such as the liver, skin, lungs, kidneys, and heart [98,99,100,101,102,103]. Wingless and the integration site (named WNT) pathway consist of a cascade of numerous signaling involved in cell development, cell metabolism, cell growth, and stem cell maintenance [104]. The WNT pathway is formed by secreted lipid-modified glycoproteins [105]. Dysregulation of the canonical WNT pathway is observed in several tumor and non-tumor diseases [106].

WNT extracellular ligands bind Frizzled (FZD) receptors, low-density lipoprotein receptor-related proteins 5 and 6 (LRP 5/6), and disheveled (DSH), which results in β-catenin accumulation and then nuclear β-catenin translocation to bind with the T-cell factor/lymphoid enhancer factor (TCF/LEF) [107]. TCF/LEF, related to nuclear β-catenin, activates the transcription of WNT target genes such as c-Myc and cyclin D1 [108].

Downregulation of the WNT/β-catenin pathway leads to the absence of binding between the extracellular WNT ligands and the FZD/LRP 5/6 complex. Thus, adenomatous polyposis coli (APC), AXIN and glycogen synthase kinase-3β (GSK-3β) form a β-catenin destruction complex and mediate the proteasomal β-catenin degradation [109]. GSK-3β inhibits the accumulation of β-catenin and its nuclear translocation [109,110].

### 4.1. Inflammation and the WNT/β-Catenin Pathway

NF-κB signaling, a key mediator of inflammation [94,111,112], is upregulated in numerous inflammatory pathways [94,112,113]. The positive interplay between NF-κB signaling and the canonical WNT/β-catenin pathway regulates the immune response and inflammation [114,115,116,117]. The activation of NF-κB increases the expression of the TCF/LEF complex and exerts an indirect positive influence on the WNT/β-catenin pathway [118]. The activation of WNT/β-catenin pathway increases NF-κB-mediated anti-apoptotic action [119,120], and stimulates inflammatory processes by stimulating β-catenin to target genes [121,122]. The activation of β-catenin/TCF4 increases the NF-κB activity in vascular smooth muscle cells [123]. Inflammation increases the production of collagen and the release of inflammatory chemokines in the development of liver fibrosis [124]. In fibrosis, CCN4, a WNT-inducible signaling pathway protein-1 (WISP1), is stimulated and leads to fibroblast proliferation and ECM synthesis [125,126]. The activation of CCN4 induces morphological transformation in skin fibrosis [127]. In hepatic fibrosis, CCl4mAb, a specific inhibitor of CCN4, decreases NF-κB activity and then downregulates the expression of pro-fibrotic factors such as TGF-β1 [128].

### 4.2. The WNT/β-Catenin and PI3K/Akt Pathways

The phosphatidylinositol 3-kinase/serine/threonine kinase (protein kinase B)/mammalian target of rapamycin (PI3K/Akt/mTOR) pathway is involved in cell growth, cell proliferation, protein synthesis, and energetic metabolism [129,130,131,132]. WNT/β-catenin pathway is an upstream activator of the PI3K/Akt/mTOR pathway [133] by inhibiting GSK-3β activity [134]. GSK-3β, a crucial inhibitor of the WNT/β-catenin pathway [135]), is a specific intracellular serin-threonin kinase involved in the regulation of numerous pathophysiological processes, such as cell membrane signaling and inflammation [136,137,138]. Moreover, in adipocyte differentiation, the activated PI3K/Akt pathway inhibits GSK-3β [139,140]. Inhibition of the PI3K/Akt pathway suppresses collagen synthesis in human and rat hepatic stellate cells [141,142] and protects against pulmonary and kidney fibrosis [143,144]. In addition, downregulation of β-catenin signaling decreases the expression of the PI3K/Akt/mTOR pathway [145,146]. The WNT/β-catenin and PI3K/Akt/mTOR pathways stimulate each other, increasing NF-κB signaling. The activation of GSK3-β also decreases NF-κB signaling [147,148].

## 5. Interactions between the TGF-β1 and Canonical WNT/β-Catenin Pathways

WNT3a, a canonical WNT ligand, activates TGF-β1 expression via β-catenin-dependent Smad2 activation, leading to the differentiation of fibroblasts into myofibroblasts [149]. Conversely, the absence of WNT ligands leads to the attenuation of TGF-β expression and fibrotic response [150]. GSK-3β activation phosphorylates Smad proteins and leads to their degradation [151].

**Figure 3:**
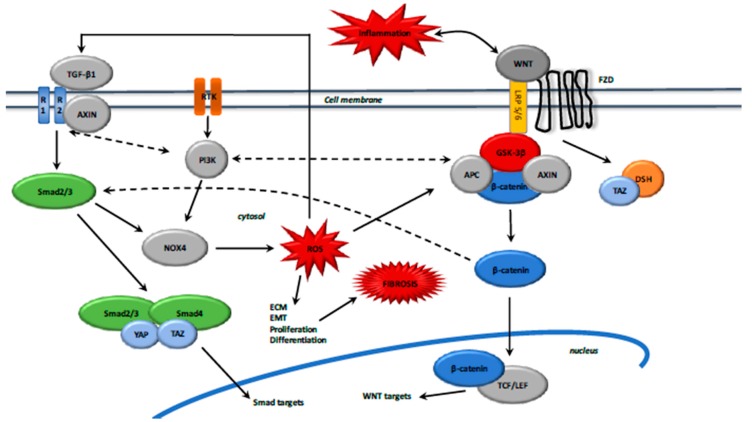
Interactions between TGF-β1 and canonical WNT/β-catenin pathway in fibrosis. Anflamation activates TGF-β1 and the WNT/β-catenin pathway. WNT activation inhibits the β-catenin destruction complex, which results in the β-catenin accumulation in the cytosol and then its translocation to the nucleus for activating WNT target genes. Upon WNT stimulation, TAZ inhibits the phosphorylation of DSH and dissociates it from the β-catenin destruction complex. The destruction complex is inhibited because YAP and TAZ dissociate from the complex. Upon TGF-β stimulation, AXIN promotes the tail-phosphorylation of Smad2/3. The activated Smad2/3-Smad4 complex associates with TAZ and YAP and then translocates to the nucleus for activating Smad targets. TGF-β1 induces Smad 2/3 and PI3K/Akt pathway activation, which in turn stimulates NOX4 activity. NOX4 enhances ROS to activate ECM, EMT, proliferation and differentation. ROS accumulation also enhances the TGF-β1 release and activation of the TGF-β1 in a positive feedback loop. TGF-β1, Transforming Growth Factor 1; PI3K, Phosphatidylinositol–4,5-biphosphatase 3-kinase; ROS, Reactive Oxygen Species; YAP, Yes-associated protein; TAZ, Transcriptional coactivator with PDZ-binding motif; DSH, Disheveled.

Moreover, activation of TGF-β also stimulates the WNT pathway by inhibiting dickkopf-related protein 1 (DKK1) expression, an inhibitor of WNT/β-catenin pathway [152]. After TGF-β and WNT stimulation, AXIN facilitates the Smad2/3 binding with a TGF-β type 1 receptor and then Smad2/3 activation [153] (Figure 3).

Upon WNT ligand activation, AXIN forms a complex with Smad7 and the E3 ubiquitin ligase Arkadia to promote the degradation of Smad7 [154], which is an inhibitor of the Smad pathway [155]. Activated Smad7 binds YAP (yes-associated protein 1) and Smurf2, which increases the affinity for TGF-β type 1 receptor and then decreases TGF-β signaling [156]. Activated Smad7 also recruits Smurf2 to induce ubiquitination and proteasomal β-catenin degradation [157].

YAP and TAZ (a transcriptional coactivator with PDZ-binding motif) mechanical signaling drives persistent fibroblast activation and sustainable fibrogenesis. Elevated YAP and TAZ levels are observed in fibrosis [158]. YAP and TAZ knockdown in cultured lung and liver fibroblasts reduces the levels of protein associated with myofibroblast differentiation, such as pro-collagen and α-SMA [159]. During fibrosis, F-actin polymerization inactivates the Hippo core kinase complex, leading to YAP and TAZ dephosphorylation and their translocation to the nucleus [15].

Stimulation by WNT3a inhibits the destruction complex because YAP/TAZ dissociates from the β-catenin destruction complex and then allows β-catenin nuclear translocation. TAZ binds DSH and dephosphorylates it upon stimulation with WNT3a. Then TAZ dissociates DSH from the destruction complex [160]. Indeed, in the absence of WNT ligands, phosphorylated YAP and TAZ bind β-catenin with activated GSK-3β and AXIN to degrade β-catenin in the proteasome [161]. In fibrosis, the activated Smad2/3–Smad4 complex is associated with YAP and TAZ for its translocation to the nucleus [162]. Crosstalk of several components of the TGF-β, WNT, and YAP/TAZ signaling plays a key role in the tuning of nucleocytoplasmic shuttling of fibrosis [15].

TAZ is also a downstream mediator of WNT signaling, independent of Hippo signaling [163]. The activation of WNT/β-catenin inhibits the phosphorylation of β-catenin and releases it from the destruction complex, which prevents TAZ degradation, resulting in the concomitant β-catenin and TAZ accumulation.

WNT/β-catenin signaling induces β-catenin and TAZ accumulation and activation of the TAZ-dependent gene responses [163].

The non-Smad pathways such as MAPK, TGF-β activated kinase (TAK1), JNK, or PI3K/Akt are also involved in interactions between TGF-β1 and canonical WNT/β-catenin pathways [164,165]. Phosphatase and tensin homolog (PTEN), a PI3K/Akt pathway inhibitor [166], inhibits the differentiation of fibroblast into myofibroblasts, as well as collagen and α-SMA expression [167].

## 6. Aerobic Glycolysis and the Fibrotic Process

The Warburg effect is involved in various tumor and non-tumor diseases [168] and occurs during the process of fibroblast differentiation into myofibroblasts [169]. The Warburg effect is described as a shift in energy production from mitochondrial oxidative phosphorylation to aerobic glycolysis despite the availability of oxygen [21].

Although aerobic glycolysis produces fewer ATP molecules than mitochondrial oxidative phosphorylation, glycolysis is much faster than oxidative phosphorylation [170]. Consequently, aerobic glycolysis may produce more total ATP than mitochondrial oxidative phosphorylation in the same amount of time [171].

Aerobic glycolysis generates the energy and metabolites involved in the regulation of cellular functions, including proliferation, extracellular matrix production, autophagy, and apoptosis [172].

In fibrosis, aerobic glycolysis provides the necessary ATP, amino acids, and nucleotides for ECM synthesis [173], and is actively involved in reprogramming cellular metabolism to enable fibroblast differentiation into myofibroblasts. Fibroblast cells support their differentiation by reprogramming nutrient uptake and metabolism. The main nutrients that myofibroblast cells use are glucose and the amino acid glutamine, which are essential to many metabolic processes involved in fibrosis.

From an energy metabolism perspective, the TGF-β1 and WNT/β-catenin pathways are central regulators of glycolytic energy metabolism in fibrosis.

Glycolysis is induced in a hypoxia-independent manner by (1) the WNT/β-catenin pathway activation via direct activation of WNT target genes, PDK1 and MCT-1, and via the stabilization of hypoxia-inducible factor 1 alpha (HIF-1α) [168,174,175,176]; and (2) TGF-β1 pathway activation via HIF-1α stabilization [16].

**Figure 4:**
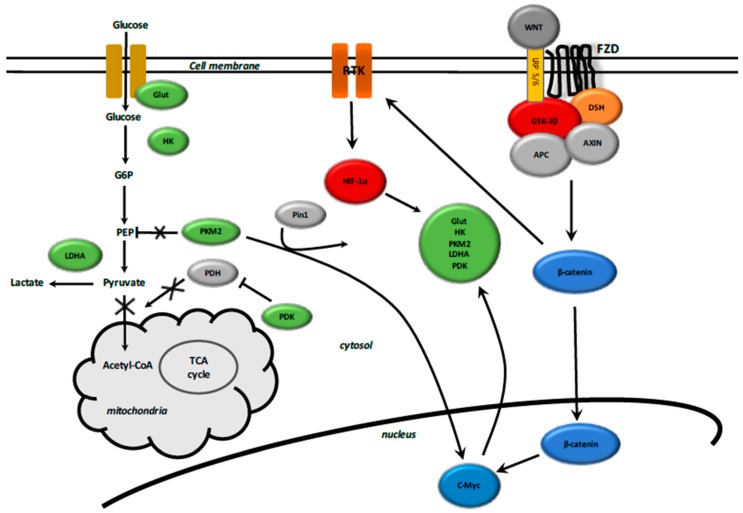
Interactions between WNT/β-catenin pathway and aerobic glycolysis. WNT ligands bind both Frizzled and LRP 5/6 receptors to initiate LRP phosphorylation of the AXIN/APC/GSK-3β complex. β-catenin phosphorylation is inhibited and this prevents its degradation in the proteasome. β-catenin accumulates into the cytosol and then translocates to the nucleus to bind TCF-LEF co transcription factors. WNT-response gene transcription is stimulated (PDK, c-Myc, cyclin D, MCT-1). MCT-1 promotes the release of lactate out of the cell. WNT/β-catenin pathway activates tyrosine kinase receptors (TKRs). Activation of PI3K/Akt increases glucose metabolism. Akt-transformed cells induce HIF-1α stabilization, which largely diminishes the glucose entry into the TCA cycle. Stimulate HIF-1α activity increases expression of glycolytic enzymes (GLUT, HK, PKM2, LDH-A). Elevated aerobic glycolysis is observed with increased production of lactate and decreased mitochondrial respiration. HIF-1α induced PDK phosphorylates PDH, which results in cytosolic pyruvate being shunted into lactate through induction of LDH-A. PDK inhibits the PDH complex into mitochondria. Thus, pyruvate cannot be fully converted into acetyl-CoA and enter the TCA cycle. c-Myc and cyclin D also activate LDH-A which converts cytosolic pyruvate into lactate. Activated PKM2 translocates to the nucleus through Pin1, then binds β-catenin and induces c-Myc expression. This activates GLUT, PKM2 and LDH-A in a positive feedback.

The TGF-β1 and WNT/β-catenin pathways activate each other in a positive feedback loop through (1) Smad signaling pathway via Smad2/3, Smad2/3/NOX4/ROS, and the Smad2/3-4 complex/YAP/TAZ, dependent on Hippo signaling; and (2) non-Smad signaling pathways such as MAPK, TAK1, JNK, and PI3K/Akt signaling (Figure 4).

HIF-1α stabilization is sustained in a hypoxia-independent manner through the molecular signaling pathways, which consist of (1) WNT/β-catenin target genes, c-Myc and cyclin D1; (2) WNT/β-catenin-induced PI3K/Akt; (3) TGF-β1-induced PI3K/Akt; (4) WNT/β-catenin-induced TAZ independent of the Hippo signaling; PKM2/β-catenin complex-induced c-Myc; and (5) the positive interplay between the TGF-β1 and WNT/β-catenin pathways.

HIF-1α is a heterodimeric nuclear transcription factor composed of two subunits: HIF-1alpha and HIF-1β. HIF-1α stabilization results in its nuclear translocation, dimerization with HIF-1β, and the transcription of genes encoding glycolysis enzymes [177]. HIF-1α undergoes proteasomal degradation after hydroxylation by HIF prolyl-hydroxylases.

Aberrant HIF signaling after HIF-1α stabilization induces energy metabolism reprogramming in the context of fibrosis by increasing glycolysis energy metabolism and decreasing the entry of glucose-derived carbons into the TCA cycle, reducing the mitochondrial oxidative phosphorylation energy metabolism [178,179]. HIF-1α represents a central regulator of glycolysis energy metabolism involved in organ fibrosis and a key player in the pathogenesis of lung fibrosis [180].

HIFs reprograms glucose and glutamine metabolism to promote the Warburg effect. HIF-1α stabilization increases glycolysis by (1) overexpression of glucose transporters (Glut) mediating increased glucose uptake and (2) activation of glycolytic enzymes such as Glut, HK2, PKM2, LDH-A, and PDK1 [180,181,182,183]. HIF activation promotes lactate production through increasing LDHA expression and decreasing the conversion of pyruvate into acetyl-CoA by upregulating PDK1, resulting in decreased activity of PDH [184,185].

PI3K/Akt signaling is activated by cytosolic β-catenin accumulation [145,146]. Activation of the PI3K/Akt pathway induces HIF-1α stabilization [183] and is associated with an increased rate of glucose metabolism [186].

Glut-1 and Glut-3 are essential for the insulin-sensitive homeostasis of glucose transport [187]. After glucose enters the cell, the final step of glycolysis is the conversion of the phosphoenolpyruvate (PEP) and ADP into pyruvate and ATP, a reaction catalyzed by the enzyme pyruvate kinase (PK). PK has four isoforms: PKM1, PKM2, PKL, and PKR. The PKM2 dimeric form has low affinity with PEP [188]. Under high glucose concentration, PKM2 is acetylated and translocated to the nucleus through the action of the peptidyl-prolyl isomerase 1 (Pin1) [189], which reduces its activity and targets PKM2 towards a lysosome-dependent degradation [190]. Nuclear PKM2 binds nuclear β-catenin and then induces c-Myc-mediated expression of glycolytic enzymes including Glut, LDH-A, PDK1, and PKM2 in a positive feedback loop, thus strengthening aerobic glycolysis [191]. Phosphorylated PKM2 levels are upregulated in fibrotic kidneys [192].

The allosteric enzyme phosphofructokinase (PFK) catalyzes the conversion from β-d-fructose-6-phosphate to β-d-fructose-1,6-biphosphate and stimulates aerobic glycolysis during fibroblast differentiation into myofibroblasts to generate sufficient energy and stabilize HIF-1α levels [180]. This conversion uses ATP and is responsible for glycolytic oscillations, leading to instabilities from which a novel state can be organized in time and space, driven by PFK with a positive feedback accountable for periodic behavior [193]. In addition, PFK has been shown to be a dissipative structure responsible for energetic metabolism behaving far from equilibrium [36,194].

The shunting of glucose carbons far from the TCA cycle results in glutamine metabolism reprogramming. The WNT target gene c-Myc stimulates glutaminolysis and drives glutamine uptake into the cytosol and the mitochondria [195]. c-Myc-activated HIF shifts from oxidative decarboxylation to reductive carboxylation of glutamine to generate citrate and acetyl-CoA for lipid synthesis [196,197,198]. c-Myc-induced glutamine enhances aspartate and nucleotide synthesis [195] by HIF-1α stabilization, which activates PDK1 [199]. Then, a minor part of the pyruvate is converted into acetyl-CoA, which enters the TCA cycle and become citrate for promoting protein and lipid synthesis.

Upregulation of glycolysis, despite an overall decrease in mitochondrial respiration, results in increased levels of the TCA cycle intermediate succinate [200]. The dicarboxylic acid transporter transfers the succinate from the mitochondria to the cytosol. Cytosolic accumulation of succinate inhibits HIF prolyl hydroxylase activity, leading to HIF-1α stabilization and activation [200]. Buildup of succinate mainly derives from glutamine-dependent anaplerosis via α-Ketoglutaric acid (αKG) and the “GABA-shunt” pathway [200]. Moreover, the lowered αKG/succinate ratio results in lowered HIF prolyl-hydroxylase activity, which uses αKG as a co-substrate for its enzyme activity and leads to HIF-1α stabilization [184,201,202]. The TCA cycle intermediate succinate is upregulated in lung myofibroblasts. Augmented glycolysis leads to increased succinate, which markedly enhances TGF-β1-induced HIF-1α expression independent of hypoxic conditions, and promotes TGF-β1-induced lung fibroblast differentiation into myofibroblasts by binding HIF-1α to the HIF-1α responsive element site within the α-SMA promoter [200].

TGF-β also induces HIF-1α stabilization and LDH5 expression via TGF-β-induced HIF-1α overexpression, which promotes glycolysis energy metabolism and lactate production through activating HIF-1α-regulated glycolytic enzymes in idiopathic pulmonary fibrosis [16]. TGF-β is abundantly present in an inactive form that requires cleavage to become biologically active [203,204,205]. TGF-β is known to be activated by heat, enzymatic cleavage, extremes of pH, integrins, and mechanical stretching [203,206,207]. TGF-β is a key cytokine responsible for fibroblast differentiation into myofibroblasts via HIF-1α overexpression. The transformation of fibroblasts into myofibroblasts that generate excess collagen and other extracellular matrix proteins ultimately leads to scar formation in idiopathic pulmonary fibrosis [208,209,210]. Moreover, the TGF-β-induced excessive production of lactic acid resulting from aerobic glycolysis in fibroblasts induces a significant decrease in the pH with acidification of the metabolic milieu, which in turn promotes activation of latent TGF-β to the fibroblast differentiation into myofibroblasts. Thus, this feed-forward loop involved in lactic acid, TGF-β, HIF-1α, and LDH strengthens aerobic glycolysis and TGF-β-induced fibroblast differentiation into myofibroblasts in idiopathic pulmonary fibrosis [16]. Stabilization of HIF-1α promotes TGF-β1-induced fibroblast differentiation into myofibroblasts in independent hypoxia signaling [169].

Hypoxia can also be critically involved in fibrosis, and stabilizes HIF-1α by inhibiting HIF prolyl-hydroxylase, which depends on oxygen as a cofactor for its enzyme activity. Thus, hypoxia causes lowered HIF prolyl-hydroxylase activity, leading to HIF-1α stabilization [178]. Thus, the fibrotic dissipative process, by switching its core metabolism from oxidative phosphorylation to glycolysis, is responsible for an energetic metabolism behaving far from the thermodynamic equilibrium [36,194].

## 7. Circadian Rhythms and Circadian Clock Genes

Numerous biological systems exhibit oscillatory behaviors such as glycolysis [211,212], or circadian rhythms [211,212,213] driven by the circadian “clock” (circadian locomotors output cycles kaput). The circadian clock, located in the hypothalamic suprachiasmatic nucleus, displays endogenous and trainable circadian rhythms of free-running periods lasting approximately 24 h.

The numerous transcription factors, responsible for the regulation of circadian rhythms, are circadian locomotor output cycles kaput (Clock), brain and muscle aryl-hydrocarbon receptor nuclear translocator-like 1 (Bmal1), Period 1 (Per1), Period 2 (Per2), Period 3 (Per3), and Cryptochrome (Cry 1 and Cry 2) [214,215]. They are subject to positive and negative self-regulation mediated by circadian rhythms [216,217]. The heterodimerization of Clock and Bmal1 enables the transcription of Per1, Per2, Cry1, and Cry2 [218]. The Per/Cry heterodimer, by translocating back to the nucleus, directly represses the Clock/Bmal1 complex and inhibits its own transcription, and thus can inhibit its activation through a negative feedback loop [218].

The Clock/Bmal1 heterodimer also activates the transcription of retinoic acid-related orphan nuclear receptors, Rev-Erbs, and retinoid-related orphan receptors (RORs). Bmal1 transcription can be activated by RORs via a positive feedback loop, whereas they can be repressed by Rev-Erbs via a negative feedback loop [218].

The imbalance of circadian rhythms is directly related to a negative feedback loop performed by a protein on its own gene expression [219,220]. Circadian rhythms are dissipative open biological systems that operate, such as to drive the fibrotic process, and work far from the thermodynamic equilibrium by exchanging spontaneously energy and matter with their surroundings, and by changing their cellular entropy production rate [11,221].

Circadian rhythms regulate numerous physiological and metabolic functions, such as heart rate, blood pressure, body temperature, sleep–awake, and feeding patterns [222]. Similarly, circadian rhythms govern energetic metabolism. Oscillation, a cellular process regulating the timing of different biological cycles leading to fibrosis, is driven by multiple irreversible cycles that hydrolyze fuel molecules such as ATP. In such biological systems, a critical amount of energy is needed to drive them to oscillate. A certain amount of energy is consumed per period to reduce the phase diffusion constant and contributes to enhancing the coherence time and phase accuracy of the oscillations. The free-energy dissipation per period, on which the phase diffusion constant depends, is proportional to the number of phase coherent periods [211].

## 8. Circadian Rhythms and Fibrosis

The fibrotic process is associated with irregular circadian phases [223,224,225]. Per2 plays a critical role in fibrosis [226,227]. *mPer2*^−/−^ mouse models develop severe liver fibrosis [226]. Per2 expression plays a protective role in hepatic fibrosis [227]. Diminution of the expression of Per2 exacerbates liver fibrosis and vascular senescence [227,228]. Bmal1-deficient fibroblasts show a dysregulation of ROS homeostasis [229]. ROS have their own circadian rhythms [230]. However, the clock-deficiency in mouse models shows low blood pressure [231], which participates in the prevention of renal fibrosis [232]. High blood pressure induces fibrosis, whereas reduction of the blood pressure prevents the fibrotic process [233,234].

ROR expression is increased during fibroblast differentiation into myofibroblasts [235]. ROR activation interacts with the WNT/β-catenin and PI3K/Akt pathways to induce mesenchymal transition [236].

### 8.1. Circadian Rhythms and TGF-β1 Signaling

Bmal1 has direct transcriptional control on components of TGF-β signaling [237]. Smad7 and Smurf2 contain putative Bmal1/Clock-binding sites [238]. Bmal1 stimulation activates TGF-β1 by activating Smad3 and inhibiting GSK-3β, and then activates fibroblast differentiation into myofibroblasts [37]. Bmal1 knockout cells show low levels of TGF-β1 and Smad proteins [238]. However, GSK-3β activation can decrease the expression of Bmal1 [239]. GSK-3β activation also decreases Smad3 and then downregulates Smad pathway and its target genes [240].

**Figure 5:**
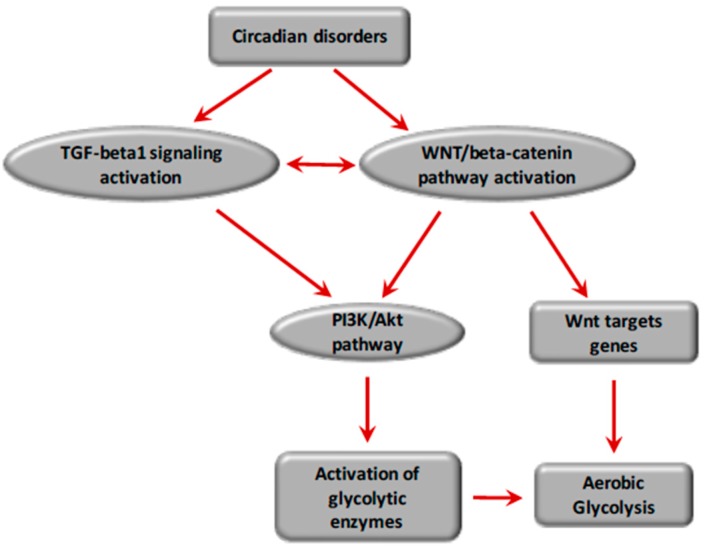
Interactions between circadian disorders and the TGF-β1 signaling and WNT/β-catenin pathway in the energetic metabolism of fibrosis. Circadiam rhytms disorders are observed in fibrosis. Dysregulation of Bmal1 induces activation of WNT pathway. PI3K/Akt pathway is activated by β-catenin accumulation and nuclear translocation. The activation of β-catenin induces transcription of WNT target genes (PDK, MCT-1, c-Myc, cyclin D, PKM2). PDK inhibits the PDH complex in mitochondria, thus pyruvate can’t be fully converted into acetyl-CoA and enter the TCA cycle. C-Myc and cyclin D activates LDH-A which converts cytosolic pyruvate into lactate. This effect is called aerobic glycolisys. Activation of WNT/β-catenin pathway induces aerobic glycolisys in fibrosis. Bmal1 stimulation activates TGF-β1 by activating Smad3 and inhibiting GSK-3β, and then activates fibroblast differentiation into myofibroblasts. PDK, pyruvate dehydrogenase kinase; APC, Adenomatous Polyposis coli; GSK-3β, glycogen synthase kinase 3 β; TCF/LEF, T-cell factor/lymphoid enhancer factor; MCT-1, Monocarboxylate Transporter 1; LDH-A, Lactat Dehydrogenase A; PI3K, Phosphatidylinositol−4,5-biphosphatase 3-kinase, PKM2, Pyruvate Kinase M2; PDH, pyruvate dehydrogenase; TCA cycle, Tricarboxylic acid cycle.

Melatonin (also named 5-methoxy-*N*-acetyltryptamine), naturally secreted by the pineal gland [241], is released during darkness, and thereby regulates sleep [242,243]. Melatonin has anti-inflammatory, antioxidant, and neuroprotective effects [242,244,245,246,247,248]. Melatonin decreases phosphorylation of GSK-3β [249,250] and can prevent pulmonary fibrosis [251,252] by inhibiting pro-fibrotic fibroblasts [253,254,255,256,257] and blocking Bmal1 expression via the repression of ROR alpha activity [258]. Furthermore, melatonin decreases the activity of Sirtuin 1 (SIRT1), which interacts with Clock to regulate Bmal1 [259,260]. SIRT1 directly inhibits the TGF-β1 pathway and induces anti-fibrotic effects [261] (Figure 5).

### 8.2. Circadian Rhythms and the WNT/β-Catenin Pathway

The RORs regulation factors act upstream of the WNT/β-catenin pathway, which possesses various putative Bmal1 clock-binding sites within its promoter. Through these interactions, circadian genes control cell cycle progression via the WNT pathway [38]. Bmal1 knockdown inhibits the expression and activity of the WNT signaling pathway [262]. Expression levels of WNT-related genes in wild-type mice are higher than those in Bmal1 knockdown mice [238,263]. Bmal1, through the canonical WNT/β-catenin activation, regulates cell proliferation and cell cycle progression [264]. Bmal1 enhances β-catenin transcription, reduces β-catenin degradation, and represses GSK-3β expression [222]. β-catenin-induced Per2 degradation involves circadian dysregulation in the intestinal mucosa of ApcMin−/+ mice [265].

In physiological conditions, the core circadian genes work in accurate feedback loops and keep the molecular clockworks in the hypothalamic suprachiasmatic nucleus. They can control peripheral clocks or cellular oscillators outside the hypothalamic suprachiasmatic nucleus [216,217]. Per1 and Per2 maintain cell circadian rhythms and regulate cell-related gene expression, such as c-Myc, to sustain the physiological cell cycle [266,267] (Figure 5).

## 9. Conclusions

Fibrosis is characterized by fibroblast proliferation and fibroblast differentiation into myofibroblasts, which generate a relaxation-free contraction mechanism associated with excessive collagen synthesis in the extracellular matrix, which promotes an irreversible tissue retraction evolving towards fibrosis. Fibrosis can occur in all tissues and tumors, including the brain.

From a thermodynamic point of view, the mechanisms leading to fibrosis are like many irreversible processes that can occur through changing the entropy production rate. The thermodynamic behavior of metabolic enzymes in fibrosis is modified by the activation of both transforming growth factor TGF-β signaling and the canonical WNT/β-catenin pathway, which leads to lower conversion of pyruvate into acetyl-coenzyme A in mitochondria and its entry into the tricarboxylic acid cycle. A major part of cytosolic pyruvate is converted into lactate as the primary alternative of oxidative phosphorylation, despite the availability of oxygen. This phenomenon, called aerobic glycolysis or the Warburg effect, generates energy and is actively involved in reprogramming cellular energy metabolism to enable the process of fibroblast differentiation into myofibroblasts.

Glycolysis is induced in a hypoxia-independent manner by (1) the WNT/β-catenin pathway activation via direct activation of WNT target genes, PDK1 and MCT-1 and via the stabilization of the hypoxia-inducible factor 1 alpha (HIF-1α), and (2) the TGF-β1 pathway activation via the HIF-1α stabilization. TGF-β1 and WNT/β-catenin pathways activate each other in a positive feedback loop through (1) the Smad signaling pathway via Smad2/3, Smad2/3/NOX4/ROS, Smad2/3-4 complex/YAP/TAZ dependent of the Hippo signaling, and (2) the non-Smad signaling pathways such as PI3K/Akt signaling.

HIF-1α stabilization is sustained in a hypoxia-independent manner through molecular pathways: (1) WNT/β-catenin target genes, c-Myc and cyclin D1; (2) WNT/β-catenin-induced PI3K/Akt; (3) TGF-β1-induced PI3K/Akt; (4) WNT/β-catenin-induced TAZ independent of the Hippo signaling; PKM2/β-catenin complex-induced c-Myc; and (5) the positive interplay between the molecular pathways TGF-β1 and WNT/β-catenin. Aberrant HIF signaling with HIF-1α stabilization induces energy metabolism reprogramming in the context of fibrosis by increasing glycolysis energy metabolism and decreasing the entry of glucose-derived carbons into the TCA cycle, thereby reducing mitochondrial oxidative phosphorylation energy metabolism. HIF-1α is a central regulator of the glycolysis energy metabolism involved in organ fibrosis, indicating a similarity between inflammation and cancer.

The molecular mechanisms of the fibrotic process, including the TGF-β1 and WNT/β-catenin pathways, can be considered dissipative structures that exchange energy or matter with their environment. They are open systems working far from the thermodynamic equilibrium and operate under a non-linear regime evolving towards non-stationary states. Indeed, circadian rhythms, which are also far-from-equilibrium open thermodynamic systems, directly drive the fibrotic process by regulating the molecular pathways TGF-β1 and WNT/β-catenin involved in the reprogramming of cellular energy metabolism, enabling the differentiation of fibroblasts into myofibroblasts and leading to the fibrotic process.
